# Adjustable single-incision mini-slings (Ajust®) versus other slings in surgical management of female stress urinary incontinence: a meta-analysis of effectiveness and complications

**DOI:** 10.1186/s12894-018-0357-0

**Published:** 2018-05-18

**Authors:** Fuding Bai, Jimin Chen, Zhewei Zhang, Yichun Zheng, Jiaming Wen, Xiawa Mao, Nan Zhang

**Affiliations:** Department of Urology, School of Medicine Hangzhou, Second Affiliated Hospital, Zhejiang University, No.88 Jiefang Road, Hangzhou, Zhejiang Province People’s Republic of China

**Keywords:** Ajust, Single-incision mini-slings, MiniArc, Transobturator slings, Meta-analysis

## Abstract

**Background:**

Adjustable single-incision mini-sling (SIMS) is a new category of SIMS for stress urinary incontinence (SUI). The aim of this study was to compare the efficacy and safety of adjustable single-incision mini-sling with other slings.

**Methods:**

Literature search in databases such as Pubmed, and Conchrane Library was performed up to December, 2015. The outcomes including cure rate, operation time, postoperative pain score and complications were reanalyzed. The pooled relative risk (RR) and mean difference (MD) with their 95% confidence interval (95% CI) were calculated by RevMan v5.0.

**Results:**

Eight studies with 1093 SUI female patients were included. There was no significant difference between adjustable SIMS and other slings (transobturator slings and MiniArc) in patients subjective cure rate and objective cure rate. In addition, adjustable SIMS was associated with a significantly shorter operative time and lower postoperative pain score when comparing adjustable SIMS with transobturator tape (MD = − 1.35; 95%CI: -2.24 to − 0.46, *P* = 0.003). For the complications, there was also no significant difference between adjustable SIMS and transobturator slings.

**Conclusions:**

Adjustable SIMS had equally efficacy for SUI compared with transobturator slings and MiniArc. However, the significantly shorter operative time and lower postoperative pain score than transobturator tape supported the clinical application of adjustable SIMS.

## Background

Based on the definition of International Continence Society, stress urinary incontinence (SUI) is the complaint of involuntary leakage of urine on effort or exertion, or on sneezing or coughing [1]. SUI is a common problem in women, which accounts for nearly 50% of all incontinent women and affects the quality of life [2]. Surgical treatment is necessary for SUI after failure of conservative treatment [3]. The midurethral sling is the mainstay of SUI treatment over the last ten years [4].Tension-free vaginal tape (TVT), which is the first generation of MUS and firstly reported by Ulmsten et al. in 1995, has been used as a standard minimally invasive procedure for SUI with a success rate of 84-95% [5]. However, it is associated with many serious complications because of the blind passage through the retropubic space, such as bladder perforation, vessel and bowel injuries, perioperative bleeding and hematoma formation [6, 7]. Subsequently, transobturator slings including tension-free vaginal tape-obturator (TVT-O) and transobturator tape (TOT), were developed with comparable cure rates and relatively less complications compared with TVT [8, 9]. However, the transobturator approaches are associated with the risk of persistent groin and thigh pain [10]. Afterwards, a new tension-free midurethral vaginal sling, which is known as single-incision mini-slings (SIMS) and the third generation of midurethral sling, is developed with the advantage of avoiding both retropubic and groin muscle trajectories [11], such as TVT-Secur and MiniArc [12]. However, a previous meta-analysis, which compared the safety and efficacy of SIMS with standard midurethral sling (SMUS, including TVT, TOT and TVT-O), did not show the superior outcomes of SIMS to SMUS [13]. Thus, it is important to perform further investigations to find a more safety and efficacious approach for treatment of SUI.

Currently, the adjustable SIMS is a new category of SIMS [14], which provides a robust insertion into the obturator internus muscle/membrane and allows post-insertion adjustment of the tape [14, 15]. Recently, some studies have been performed for comparing the adjustable SIMS with other slings [16]. However, it still cannot determine whether the efficacy and safety of adjustable SIMS are superior to these slings due to the small sample size in single study and inconsistent results among these studies. Therefore, we performed this meta-analysis to comprehensively evaluate the efficacy and safety of adjustable SIMS comparing with other slings.

## Methods

### Literature search

This meta-analysis was conducted according to the Preferred Reporting Items for Systematic Reviews and PRISMA statement guidance [17]. The studies were searched in Medline, Embase, Pubmed, EBSCO, Conchrane Library, and Science, up to December, 2016, using the following key words: “Ajust” or “adjustable” and “urinary incontinence” or “stress urinary incontinence” and “female”. No language restriction was applied.

### Selection criteria

The studies included in this meta-analysis should meet the following criteria: (1) the study type was randomized controlled trial (RCT); (2) participants were females over 18 years old and diagnosed with SUI; (3) the studies investigated the primary SUI surgery and compared adjustable SIMS with other slings; (4) the outcomes such as operation time, postoperative pain score, postoperative complications, and/or patients subjective cure rate and objective cure rate were reported.

The studies were excluded according to the following criteria: (1) there was no available data for meta-analysis; (2) they were reviews, letters and comments. In addition, for the duplicated publications, only the one with the most complete data was included.

### Data extraction and quality assessment

The literature search and data extraction were performed independently by two authors, and the disagreements were resolved by discussion or consulting a third reviewer. The following data were recorded from each study: first author’s name; country, sample size, type of other slings, follow up, definition of patients subjective cure, definition of objective cure, as well as the clinical outcomes.

The Jadad score system was used to assess quality of the included studies [18]. There were three items in the score system and each item had one or two questions: randomization (two questions: was the study randomized? was the randomization method described and appropriate?), blinding (two questions: was the study described as double blind? was the method of blinding described and appropriate?) and description of withdrawals and dropouts (one question: was there a description of withdrawals and dropouts?). One score was assigned for each “yes” answer to each question. The studies with 3-5 scores were regarded as high-quality, while those with 0-2 scores were low-quality.

In addition, risk of bias of each study was assessed by the Cochrane Collaboration’s tool [19].

### Statistical analysis

Data were analyzed using RevMan v5.0. The relative risk (RR) and mean difference (MD) with their 95% confidence interval (95%CI) were used as the effect size to assess the effectiveness and complications of adjustable SIMS versus other slings. A Z-test was used to test the significance of RR and MD. *P* < 0.05 was considered statistically significant. The heterogeneity among studies was measured using the Cochran’s Q-statistic and I^2^ test. A significant Q-statistic (*P* < 0.10) or I^2^-statistic (I^2^ > 50%) indicated significant heterogeneity across the studies, and then the random effects model was used to pool the data. Otherwise, the fixed effects model was applied.

## Results

### Characteristics of included studies

**Fig. 1:**
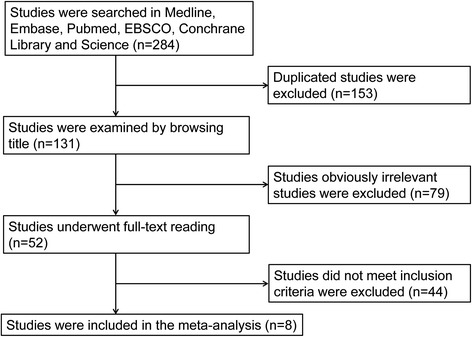
Flow chart of study selection in the meta-analysis

Initially, 284 articles were retrieved from the databases. After removing the duplicates, total 131 articles were remained. Subsequently, 79 obviously irrelevant studies were excluded by scanning the titles. Then, 44 articles were excluded by reviewing the full-texts and abstracts according to the inclusion and exclusion criteria. Finally, a total of 8 studies [16, 20–26] were included in the meta-analysis (Fig. 1).

**Fig. 2:**
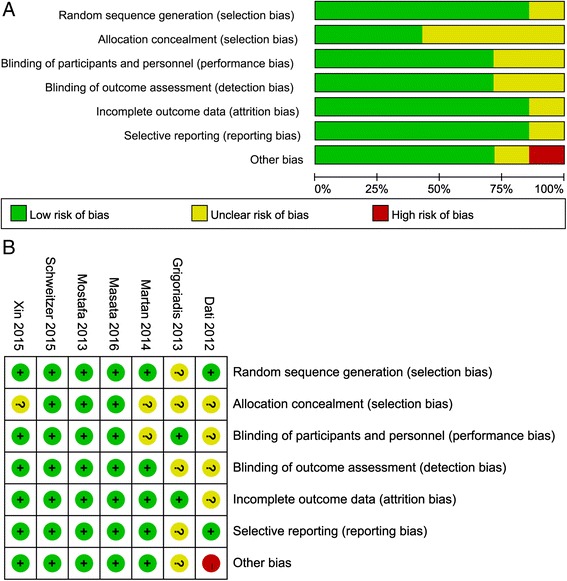
Risk of bias assessed by the Cochrane Collaboration’s tool. **a** Methodological quality item of all included studies; **b** Methodological quality item for each included study. +: low risk of bias; “?”: unclear risk of bias; “-”: high risk of bias

As shown in Table 1, all the RCTs were published between 2012 and 2016. Based on the Jadad score system, five studies were identified as high quality and two were low quality. In addition, a majority of the studies had a low risk of bias (Fig. 2), indicating a high quality of the included studies. Totally, 1093 females with SUI were included in this meta-analysis. There were differences in definitions of patients subjective cure rate and objective cure rate among the eight studies.

**Table 1:** Characteristics of included studies

First author, year	Region	Sample size		Age	Follow up (months)	Definition of subjective cure	Definition of objective cure	Outcomes	Jadad score
Xing X, 2016	China	Adjustable SIMS	184	57.6 ± 6.8	12	Defined as “very much improved” or “much improved” on the PGI-I scale	Negative CST	(a) (b) (c)	3
		TVT-O	184	56.5 ± 5.7					
Mostafa, 2012 and 2013	UK	Adjustable SIMS	69	52.6 ± 11.2	4-6/12^a^	Responses of “very much/much improved” on PGI-I	Negative CST with comfortably full bladder	(a) (b) (c) (d)	3
		TVT-O	68	49.4 ± 8.8					
Grigoriadis, 2013	Greece	Adjustable SIMS	86	65.2 (47-81)	22.3 (12-36)	No loss of urine with exercise, coughing or weight lifting	Absence of stress urinary incontinence during CST	(a) (b) (c) (d)	3
		TVT-O	85	67.2 (49-82)					
Masata, 2016	Czech	Adjustable SIMS	50	55.8 ± 10.2 58.9 ± 12.4	12	No stress leakage of urine after surgery based on the responses to the ICIQ-UI SF	Negative CST	(a)(a)(b)(c)	4
		TVT-O	50						
Dati, 2012	Italy	Adjustable SIMS	57	NA	6	NA	Negative CST	(b) (c)	3
		TVT-O	57						
Schweitzer, 2015	Netherland	Adjustable SIMS	92	50.86 ± 9.6	12	A negative answer to the Urogenital Distress Inventory question	Negative CST at a bladder volume of at least 300 mL	(a) (b) (c)	4
		TOT	48	48.36 ± 10.2					
Martan, 2014	Czech	Adjustable SIMS	31	58.97 ± 8.40	28	A drop in the score of more than 50% compared to before the operation based on ICIQ-SF	Negative CST at a bladder volume of at least 300 mL	(a) (a) (b)	2
		MiniArc SIMS	32	60.19 ± 8.12	29				

*SIMS* Single-incision mini-slings, *TVT-O* Tension-free vaginal tape-obturator, *TOT* Transobturator tape, *PGI-I* Patient global impression of improvement, *ICIQ-SF* International Consultation on Incontinence Questionnaire – Short Form, *SUI* Stress urinary incontinence, *CST* Cough stress test, *NA* Not applicable. Outcomes:(a) subjective cure rate, (b) objective cure rate, (c) operative details (such as operation time postoperative pain score) and postoperative complications, (d) sexual function. ^a^the subjective and objective cure rate at 12 months follow up in the study of Mostafa 2013 was used in this meta-analysis; the operative details and postoperative complications at 4-6 months follow up in the study of Mostafa 2012 was used for meta-analysis. ^b^the study of Palomba et al. published in 2012 reported the operative details and postoperative complications and the study of Palomba et al. published in 2014 reported the data of patients subjective and objective cure rate

### Comparison of subjective cure rate and objective cure rate for treating SUI patients

**Fig. 3:**
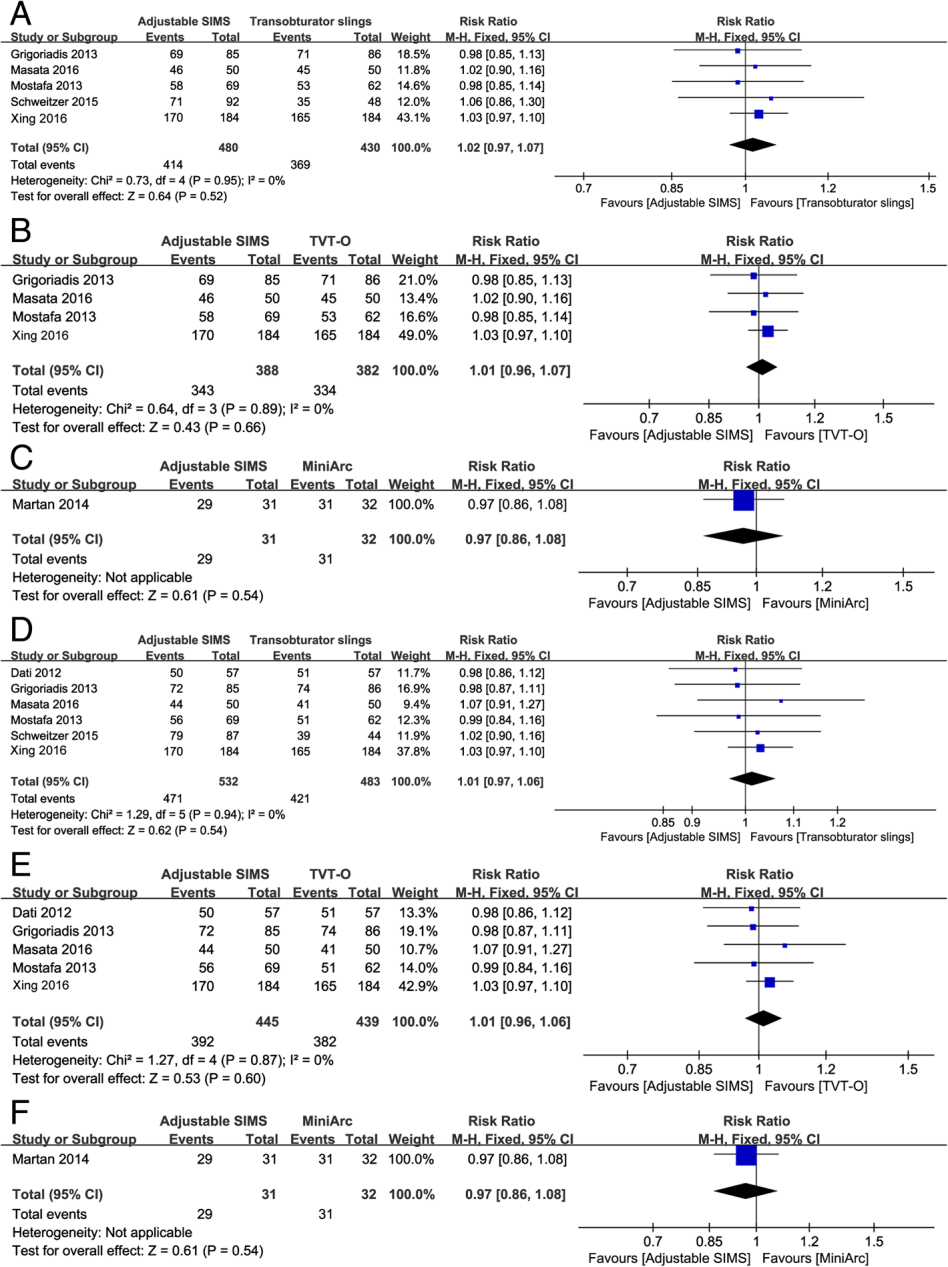
The forest plots of meta-analysis for patients subjective cure rate and objective cure rate. **a** patients subjective cure rate of adjustable SIMS versus transobturator slings; **b** patients subjective cure rate of adjustable SIMS versus TVT-O; **c** patients subjective cure rate of adjustable SIMS versus MiniArc; **d** objective cure rate of adjustable SIMS versus transobturator slings; **e** objective cure rate of adjustable SIMS versus TVT-O; **f** objective cure rate of adjustable SIMS versus MiniArc

When comparing adjustable SIMS with transobturator slings or TVT-O alone, as no significant heterogeneity (I^2^ = 0%, *P* > 0.1) was detected among the included studies for patients subjective cure rate and objective cure rate, thus the fixed effects model was used to combine the data. The pooled estimates showed that there was no evidence of significant differences in patients subjective cure rate (RR = 1.02, 95%CI: 0.97 to 1.07, *P* = 0.95, Fig. 3a) and objective cure rate (RR = 1.01, 95%CI: 0.97 to 1.06, *P* = 0.94, Fig. 3d) between patients received adjustable SIMS and transobturator slings. Likewise, no significant differences between adjustable SIMS and TVT-O alone were detected (patients subjective cure rate: RR = 1.01, 95%CI: 0.96 to 1.07, *P* = 0.89, Fig. 3b; objective cure rates: RR = 1.01, 95%CI: 0.96 to 1.06, *P* = 0.87, Fig. 3e).

When comparing adjustable SIMS with MiniArc SIMS, significant heterogeneity between the two studies was found, so the random effects model was applied. Similarly, there was also no significant difference between adjustable SIMS and MiniArc SIMS in patients subjective cure rate (RR = 0.97, 95%CI: 0.86 to 1.08, Fig. 3c) and objective cure rate (RR = 0.97, 95%CI: 0.86 to 1.08, Fig. 3f).

### Comparison of operation details

**Fig. 4:**
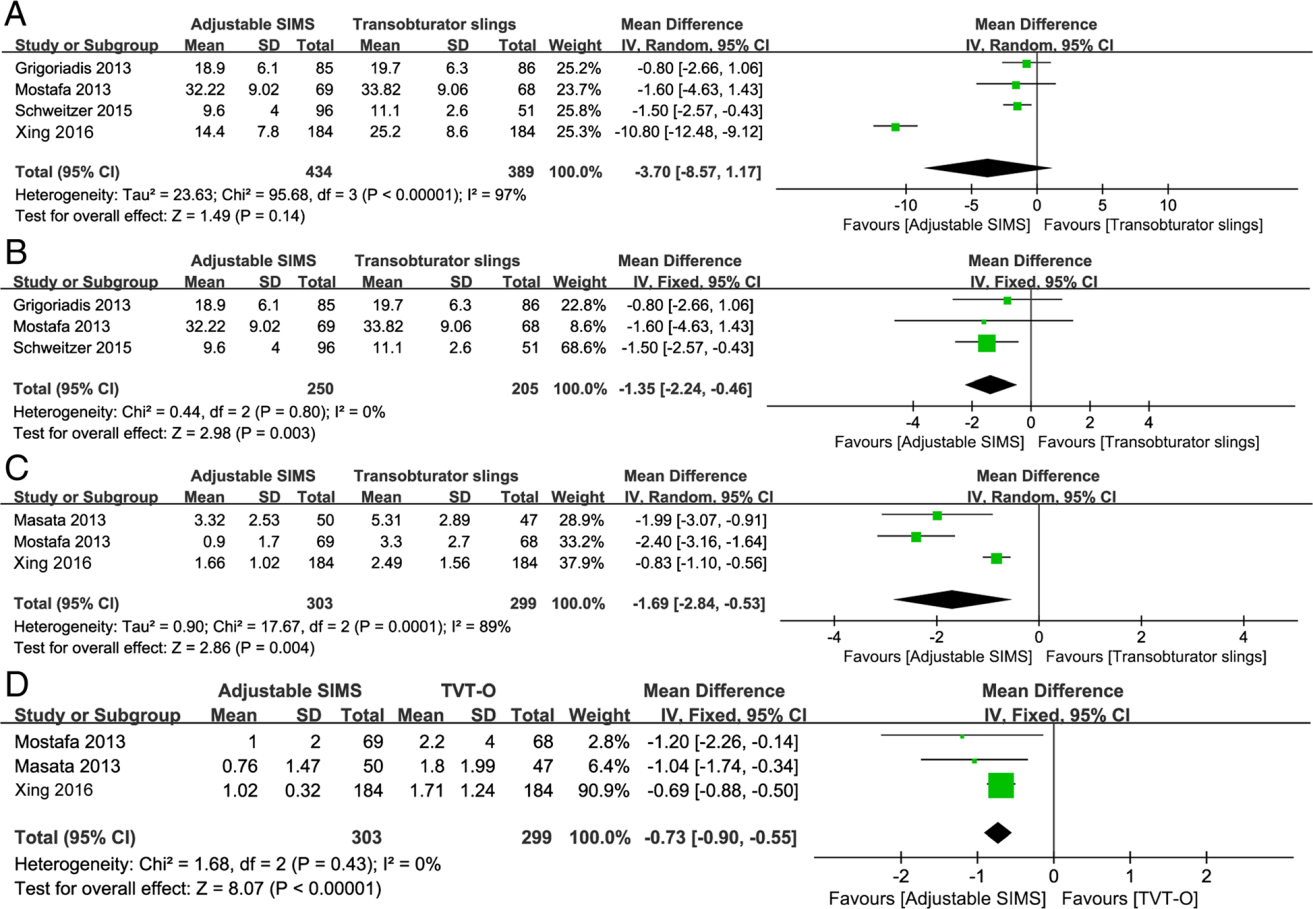
The forest plots of meta-analysis for operation details. **a** operation time of adjustable SIMS versus transobturator slings; **b** operation time of adjustable SIMS versus TVT-O; **c** comparison of adjustable SIMS versus TVT-O in postoperative pain score at the first day after operation; **d** comparison of adjustable SIMS versus TVT-O in postoperative pain score at the fourth day after operation

For operation time, there was significant heterogeneity (I^2^ = 97%, *P* < 0.00001) among the three included studies. Thus, the random effects model was applied. The pooed estimate indicated that the adjustable SIMS had a shorter operation time than transobturator slings (MD = − 3.70; 95%CI: -8.57 to 1.17, *P* = 0.14, Fig. 4a) but without significance. However, after excluding the study of Xing et al. which reported the comparison between adjustable SIMS and TOT-V, the significant difference was detected (MD = − 1.35; 95%CI: -2.24 to − 0.46, *P* = 0.003, Fig. 4b) under a fixed effects model.

In addition, the postoperative pain score by visual analog scale was investigated in three of the included studies [24–26], which compared the adjustable SIMS and transobturator slings. However, the data in the study Schweitzer et al. (which reported the comparison between adjustable SIMS and TOT) were unavailable for meta-analysis [22], so this meta-analysis only assessed postoperative pain score of adjustable SIMS comparing with TVT-O. Significant heterogeneity (I^2^ = 89%, *P* = 0. 0001) was found among the three studies for the postoperative pain score, so the random effects model was used. The pooled results showed that patients received adjustable SIMS had significantly lower postoperative pain scores than those received TVT-O at the first (MD = 1.69, 95%CI: -2.84 to 0.53, *P* = 0.0001, Fig. 4c) and fourth (MD = − 0.73, 95%CI: 0.90 to − 0.55, *P* < 0.0001, Fig. 4d) day after operation.

### Comparison of complications

**Fig. 5:**
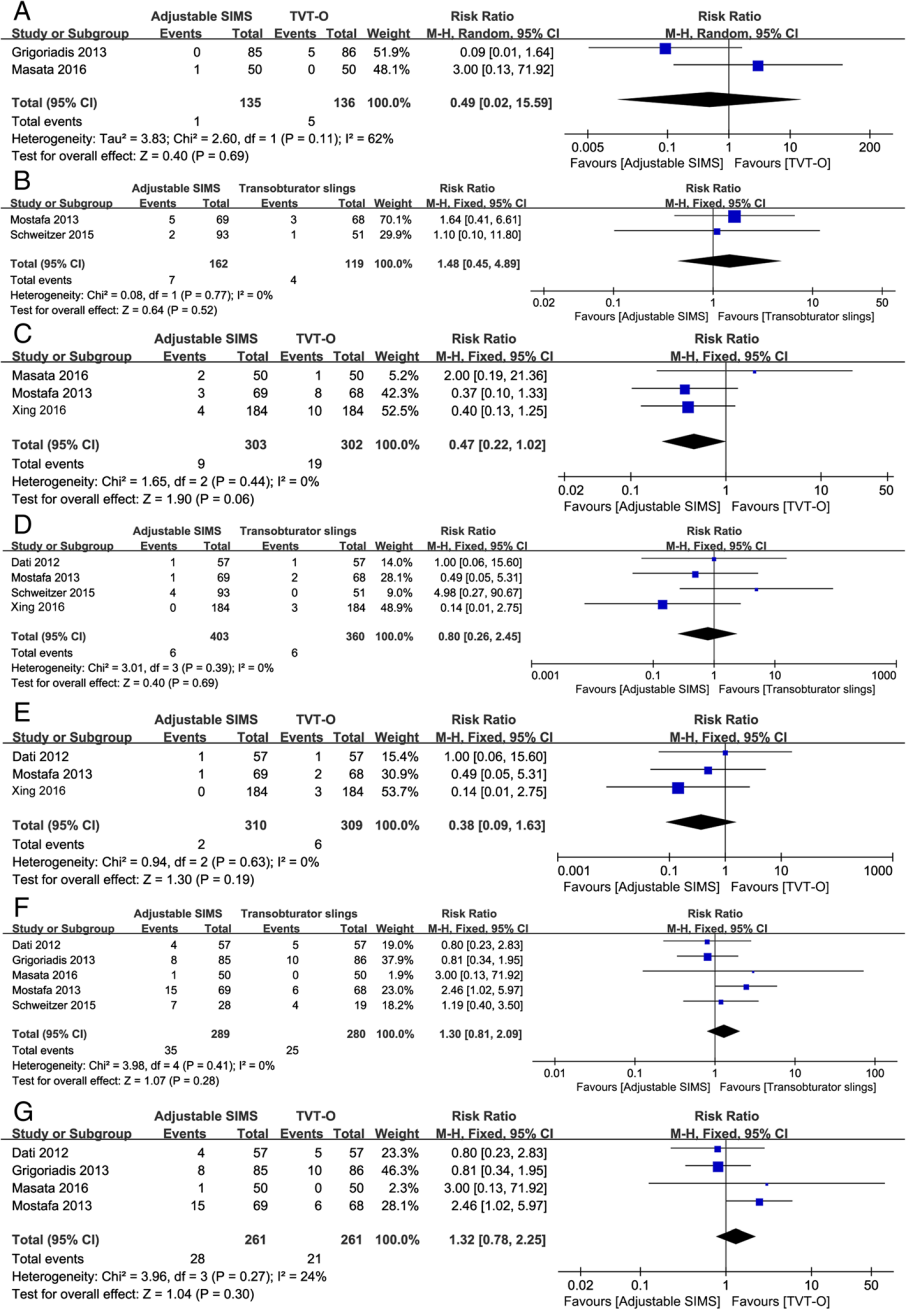
The forest plots of meta-analysis for postoperative complications. **a** comparison of adjustable SIMS versus TVT-O in groin pain; **b** comparison of adjustable SIMS versus transobturator slings in repeated continence surgery; **c** comparison of adjustable SIMS versus TVT-O in postoperative voiding difficulties; **d** comparison of adjustable SIMS versus transobturator slings in vaginal tape erosion; **e** comparison of adjustable SIMS versus TVT-O in vaginal tape erosion; **f** comparison of adjustable SIMS versus transobturator slings in de novo urgency and/or worsening of preexisting surgery; **g** comparison of adjustable SIMS versus TVT-O in de novo urgency and/or worsening of preexisting surgery

The postoperative complications were also reanalyzed in this meta-analysis. No significant heterogeneity (I^2^ < 50%, *P* > 0.1) was found among the included studies for rates of repeated continence surgery, postoperative voiding difficulties, vaginal tape erosions and de novo urgency and/or worsening of preexisting urgency, so the fixed effects model was used to pool the data. However, random effects model was used to combine the data of groin pain due to significant heterogeneity (I^2^ = 62%, *P* = 0.11). The pooled estimates demonstrated that there was no significant difference between patients receiving adjustable SIMS and transobturator slings in the rates of repeated continence surgery (RR = 1.48, 95%CI: 0.45 to 4.89, *P* = 0.52, Fig. 5b), vaginal tape erosions (RR = 0.80, 95%CI: 0.26 to 2.45, *P* = 0.69, Fig. 3d) and de novo urgency and/or worsening of preexisting urgency (RR = 1.30, 95%CI: 0.81 to 2.09, *P* = 0.28, Fig. 5f). Moreover, results also showed that compared to the patients received TVT-O, the patients received adjustable SIMS had a similar incidence of groin pain (RR = 0.49, 95%CI: 0.02 to 15.59, *P* = 0.69, Fig. 5a), postoperative voiding difficulties (RR = 0.47, 95%CI: 0.22 to 1.02, *P* = 0.06, Fig. 5c), vaginal tape erosions (RR = 0.38, 95%CI: 0.09 to 1.63, *P* = 0.619, Fig. 5e) and de novo urgency and/or worsening of preexisting urgency (RR = 1.32, 95%CI: 0.78 to 2.25, *P* = 0.30, Fig. 5g).

## Discussion

In this meta-analysis, we comprehensively compared the effectiveness and complications of adjustable SIMS and conventional slings for treating SUI patients. Reportedly, the efficacy of SUI correction is limited in those patients undergoing pelvic reconstructive surgery and corrective operation of pelvic organ prolapse is ineffective for 74.4% SUI [27], and previous continence surgery is independent risk factors for the lower success rate of TVT for SUI correction. Thus, in order to avoid the factor such as prior surgeries, concomitant prolapse associated, and surgical correction of prolapse to impact the assessment outcomes, we mainly focused on the primary SUI in females.

In our study, we found that the primary SUI patients received adjustable SIMS did not have superior outcomes to the primary SUI patients received other slings (including transobturator slings, TVT-O and MiniArc) in patients subjective cure rate and objective cure rate. The possible explain for the no obvious difference outcomes of subjective and objective cure rates between adjustable SIMS and conventional slings groups were the same treatment principle for the surgical correction of SUI in female patients. These results were in line with the two previous meta-analyses [13]. Compared with the meta-analysis of Mostafa et al. [13], we especially concerned the adjustable SIMS including more relevant studies. Moreover, the differences in operation details and complications between adjustable SIMS and other slings were investigated in this study. By contrast, only the differences in patients subjective cure rate and objective cure rate between adjustable SIMS and SMUS was assessed in the meta-analysis of Mostafa et al. Compared with the meta-analysis reported by Zhang et al. [28], we specially analyzed the comparison between adjustable SIMS and TVT-O as well as between adjustable SIMS and MiniArc SIMS. In addition, the events of patients subjective cure rate and objective cure rate were recorded conversely in the two groups between one of the included studies of Zhang et al. and that meta-analysis [20, 28].

Based on the above mentioned, no obvious difference outcomes of subjective and objective cure rates between adjustable SIMS and conventional slings groups were found. Notably, it is reported that compared with conventional slings, the adjustable SIMS is a minimally invasive technique with less slings length and mesh material to reducing the foreign body in the patient’s body [29].

Our results also showed that the patients underwent adjustable SIMS had a shorter operation time than the patients underwent transobturator slings but without significance. However, after excluding Xing’s study that compared adjustable SIMS with TOT-V, the difference was significant. The change of results after excluding the study involving comparison of adjustable SIMS and TOT-V indicated that the operation time in adjustable SIMS may be significantly shorter than TOT but not TVT-O. The potential reason may be that the outside to inside technique in TOT takes more time than inside to outside in TVT-O. More studies were required to verify this speculation. In addition, the different measurement scheme of operation time among the included studies (such as the time from incision to its closure and the overall time spent in the operating theatre) may affect the results. Thus, more studies with larger sample size and unified measurement scheme should be performed to confirm the results of this study.

A previous study has confirmed that patients received SIMS significantly improved the postoperative pain profile than transobturator slings [21]. Consistent with this study, we found the postoperative pain score in patients received adjustable SIMS was significantly lower than that in patients received TVT-O. Although no available data of postoperative pain could be used for this meta-analysis in the study of Schweitzer et al., it also reported the lower early postoperative pain scores of adjustable SIMS than TOT [22]. These results indicated that, similar to SIMS, the adjustable SIMS also had the advantage of lower postoperative pain score than transobturator slings. Thus, the lower postoperative pain score of adjustable SIMS may be caused by the characteristics of SIMS. In SIMS, a single vaginal insertion approach was utilized to avoid the blind passage of the trochars through the retropubic area and the groin/adductor muscles. The single vaginal insertion approach may be the main reason resulting in the lower postoperative pain score in SIMS and adjustable SIMS. However, the different anesthesia protocols among studies may affect the results of early postoperative pain score and further studies should consider this influence. Besides, Palomba et al. reported that there was no significant difference in postoperative pain score among the three SIMSs [30]. However, only this one study reported the comparison of adjustable SIMS and other SIMSs in postoperative pain [30], so the meta-analysis could not be performed. More studies were required to further investigate the postoperative pain in adjustable SIMS comparing with other SIMSs.

In addition, the analysis for complications indicated no significant difference between adjustable SIMS and transobturator slings or TVT-O alone. A previous meta-analysis reported that the SIMS was associated with the higher repeated continence surgery rates [13]. Moreover, the recent meta-analysis also found a nonsignificant trend of higher repeated continence surgery rates in patients received SIMS [28]. Considering the results in this meta-analysis (adjustable SIMS had similar repeated continence surgery rate to transobturator slings), we inferred that the adjustable SIMS might have less rates of repeated continence surgery than other SIMSs. More studies were required to prove this speculation.

Some limitations should be noted in this study. Firstly, the sample size and numbers of included studies were small. Secondly, the evaluation of publication bias was not assessed due to less than 10 included studies. Third, the heterogeneity was found in this study, the differences in race of participants, definitions of cure rate and types of transobturator slings may be the heterogeneity sources. However, subgroup analysis could not be performed to explore the heterogeneity sources due to inadequate data. Thus, these confounding factors may provide bias for the results of meta-analysis. Fourth, the follow up duration in these included studies were different and not long enough, thus more studies with long term follow up were required to further confirm the efficacy of adjustable SIMS. Fifth, we failed to compare all the indicators used in this meta-analysis between the adjustable SIMS and each slings method due to little involved studies was found. In addition, only two studies investigated the comparison between adjustable SIMS and other SIMSs. The comparison between adjustable SIMS and the TVT-Secur SIMS was not assessed due to only one study involving TVT-Secur SIMS. Thus, more RCTs with larger sample size and longer term follow up were required to further investigate the efficacy and safety of adjustable SIMS comparing with other slings, especially the other SIMSs.

## Conclusions

This meta-analysis showed that the adjustable SIMS was as effective as transobturator slings and MiniArc in curing primary SUI patients in female. In addition, the adjustable SIMS was recommended due to shorter operative time and lower postoperative pain than TOT and TVT-O, respectively. However, the efficacy of adjustable SIMS approach needed to be further verified using multicenter, large sample, and long-term follow-up studies.

## Abbreviations

95% CI: Confidence interval
MD: Mean difference
RCT: Randomized controlled trial
RR: Relative risk
SIMS: Single-incision mini-sling
SIMS: Single-incision mini-slings
SUI: Stress urinary incontinence
TOT: Transobturator tape
TVT: Tension-free vaginal tape
TVT-O: Tension-free vaginal tape-obturator
